# Considering quality of care for young adults with diabetes in Ireland

**DOI:** 10.1186/1472-6963-13-448

**Published:** 2013-10-29

**Authors:** Myles Balfe, Ruairi Brugha, Diarmuid Smith, Seamus Sreenan, Frank Doyle, Ronan Conroy

**Affiliations:** 1 Department of Public Health Medicine and Epidemiology, Royal College of Surgeons in Ireland, St. Stephen’s Green, Dublin, Ireland; 2 Department of Sociology, University College Cork, Cork, Ireland; 3 Department of Psychology, Royal College of Surgeons in Ireland, St. Stephen’s Green, Dublin, Ireland; 4 Endocrinology Department, Beaumont Hospital, Dublin, Ireland; 5 Endocrinology Department, Connolly Hospital, Dublin, Ireland

**Keywords:** Type 1 diabetes, Quality of care, Young adult, Emerging adult, Ireland

## Abstract

**Background:**

Research on the quality of diabetes care provided to young adults with Type 1 diabetes is lacking. This study investigates perceptions of quality of care for young adults with Type 1 diabetes (23–30 years old) living in the Republic of Ireland.

**Methods:**

Thirty-five young adults with Type 1 diabetes (twenty-nine women, six men) and thirteen healthcare professionals (ten diabetes nurse specialists, three consultant Endocrinologists) were recruited. All study participants completed semi-structured interviews that explored their perspectives on the quality of diabetes services in Ireland. Interviews were analyzed using standard qualitative thematic analysis techniques.

**Results:**

Most interviewees identified problems with Irish diabetes services for young adults. Healthcare services were often characterised by long waiting times, inadequate continuity of care, overreliance on junior doctors and inadequate professional-patient interaction times. Many rural and non-specialist services lacked funding for diabetes education programmes, diabetes nurse specialists, insulin pumps or for psychological support, though these services are important components of quality Type 1 diabetes healthcare. Allied health services such as psychology, podiatry and dietician services appeared to be underfunded in many parts of the country. While Irish diabetes services lacked funding prior to the recession, the economic decline in Ireland, and the subsequent austerity imposed on the Irish health service as a result of that decline, appears to have additional negative consequences. Despite these difficulties, a number of specialist healthcare services for young adults with diabetes seemed to be providing excellent quality of care. Although young adults and professionals identified many of the same problems with Irish diabetes services, professionals appeared to be more critical of diabetes services than young adults. Young adults generally expressed high levels of satisfaction with services, even where they noted that aspects of those services were sub-optimal.

**Conclusion:**

Good quality care appears to be unequally distributed throughout Ireland. National austerity measures appear to be negatively impacting health services for young adults with diabetes. There is a need for more Endocrinologist and diabetes nurse specialist posts to be funded in Ireland, as well as allied health professional posts.

## Background

Young adults with Type 1 diabetes are a high-risk group in that their diabetes control is often sub-optimal and they have an increased mortality risk compared to the general population [1-4]. International studies have noted that young adults with Type 1 diabetes are between three and six times more likely to die than their age-matched peers, most commonly from acute metabolic complications [1,5,6]. Furthermore, a subset of these young people begin to develop serious long-term diabetes complications during young adulthood [1]. Over a third of young adults with Type 1 diabetes report depressive symptoms [4,7].

Healthcare systems across the world have responded to young adults’ risky physical and mental health statuses by developing specialist services for them, for example specialist young adult clinics [3,4]. The aim of these services is to improve the quality of care that is offered to young adults with diabetes, thereby encouraging them to engage with health services and motivating them to improve their control. Although these services sometimes have mixed results, the importance of providing high quality care to young adults with diabetes is now widely recognized [4].

Most research that has been conducted on young adults with diabetes has focused on young adults in the first phase of young adulthood – those who are in their late teens and early twenties [7,8]. This is considered to be the riskiest developmental period for young adults with diabetes [4,8,9]. The majority of this research e.g. [6,7] examines what the health services researcher Avedis Donabedian refers to as clinical and psychosocial *outcomes*, particularly the effects of transitional care programmes on young adults’ health statuses [1,10,11]. However some diabetes researchers [3,8,10] have also explored what Donabedian refers to as structural and process aspects of young adults’ care. *Structure*, as used by Donabedian, refers to the organizational characteristics of health systems, principally comprising two components: the healthcare workers who make up the health system (e.g. numbers, professional types, their qualifications, etc.), and the physical materials and resources (facilities, equipment, money) and organisational structures through which they provide care. *Process* refers to all of the actions that contribute to patient-care and includes such concepts as continuity of care, quality and quantity of interpersonal interactions between patients and professionals. Those diabetes researchers who have investigated structural and process aspects of care have noted that young adults place a great deal of importance on establishing relationships with healthcare professionals, and on the quality of the emotional ‘atmosphere’ through which their care is provided [3,8,10].

While diabetes researchers and clinicians have concentrated most of their efforts on the late teens and early twenties, they also recognise a second phase of young adult development [1,4]. This latter developmental phase broadly extends from about 23–30 years of age. It is a period where young adults experience a maturing sense of identity and begin to assume adult roles in society [4]. It is a phase characterised by stability, whereas the first phase of young adulthood is characterised by risk and transition. In general, specialist services are not in-place for young adults in this age range (particularly those between twenty-five and thirty years of age) and little research has been published on the quality of care that is offered to them. As a result, although the provision of good quality care during this latter developmental period may have significant long-term benefits, researchers and clinicians know little about whether these ‘older’ young adults are in fact receiving good quality care [4,12]. This is an important informational absence given the that uneven quality of care is often identified in relation to individuals with diabetes [13-16].

The present article therefore aims:

•To investigate young adults’ (23–30 years of age) and healthcare professionals’ perceptions and experiences of the quality of diabetes care that is offered to young adults in Ireland.

•To determine if there is inequity in the quality of care that is provided to young adults with diabetes;

•To explore perceptions around how the health insurance status impacts on access and care.

•To explore young adults and healthcare professionals’ levels of satisfaction with Irish health services for young people with diabetes.

•To examine if recent funding cuts to the Irish health system as a result of government austerity measures appear to be impacting on health services for these young people.

## Methods

### Rationale for qualitative approach

This study used a qualitative methodology. The principle reason for this was that we wanted to explore in detail young adults’ perceptions and experiences of diabetes services, rather than the outcomes of young adults’ utilization of such services. Researchers have recently begun to argue for the importance of learning directly from patients when assessing quality of care, and relying less on the concepts and measures derived from the perspectives of clinicians and researchers [17,18]. Qualitative approaches are particularly useful for exploring patients’ views in areas that have been previously understudied, such as healthcare quality for young adults with diabetes in their twenties, and can act as prerequisites to undertaking quantitative measurements in larger representative samples [19].

### Sampling

Young adults (n=32) were primarily recruited from an Irish Facebook support group for young adults with Type 1 diabetes, and from the Facebook page of Diabetes Ireland, Ireland’s leading diabetes charity. The recruitment message indicated that all young adult interviewees would be offered a 30 Euro gift token for their time. We concentrated our recruitment efforts on Facebook because we felt that the social media site would provide an efficient and cost-effective platform for reaching a young adult audience. Facebook recruitment advertisements indicated that the project was looking to talk to young adults with Type 1 diabetes who were between 23 and 30 years of age. Thirty of the thirty-two young adults who responded to the advertisements and were interviewed attended specialist diabetes centers, predominantly in Dublin, Cork and Galway cities, though half of these had attended non-specialist, rural settings in the past for their diabetes care. We stopped recruiting young adults after interview thirty-two as we felt that we had reached data saturation, that is the point where no new themes were emerging from the interviews. To ensure that this was in fact the case we completed an additional three interviews with young adults of a similar age who we recruited from a specialist young adult clinic in a Dublin hospital. The themes that arose in those three interviews were similar to those that arose in the Facebook interviews and therefore we did not seek to recruit more participants, i.e. we were confident that we had reached data saturation.

Twenty-nine women and six men with Type 1 diabetes were interviewed in all. The overall sample size (35 young adults) meets best practice guidelines for studies based on semi-structured qualitative interviews [20]. Young adults’ mean age was 26.9. Their mean A1c was 7.95, SD=.76. 25.7% of them were on an insulin pump. 94% were educated to at least degree level and 64% had private health insurance.

Thirteen healthcare professionals were also interviewed, three consultant endocrinologists (all male, and all working in specialist urban teaching hospitals) and ten diabetes nurse specialists (all female, eight working in specialist diabetes centres and two in general rural hospital settings). We did not supply professionals with gift cards because of cost reasons. Each healthcare professional who was interviewed was the clinical lead/coordinator for young adults with diabetes in their hospital setting. At the time that the study was conducted there were eighteen specialist diabetes centres in Ireland of which representatives of eleven of those centers were included in the study. Healthcare professionals were interviewed primarily so that we could capture their perspectives on quality of care for young adults with diabetes in Ireland. We felt that professionals would be able to give a broader overview of Irish diabetes services than the young people who might only have experiences of one or two settings. Also, we felt that it would be interesting to compare and contrast young adults’ and professionals’ perspectives in order to determine if professionals identified the same quality of care issues as young adults did.

### Approach

Interviews were chosen as the research method because they would enable us to explore interviewees’ accounts in detail and because interviewees were located in disparate regions of Ireland. As such it would not have been possible to convene focus groups with the sample that we recruited.

All interviews were conducted by the first author who, at the time that the study was conducted, was thirty-one years of age. Prior to the study commencing the first author had conducted 220 semi-structured interviews on previous research projects. Seventeen of the young adult interviews were conducted over the telephone and eighteen in person. Interviews with young adults lasted 34–86 minutes, with telephone interviews being shorter. Interviews with healthcare professionals were most often conducted over the telephone (n=11) and lasted approximately twenty minutes. The reason for the shorter length of the professionals’ interviews vis-a-vis the young adults is that we usually interviewed professionals between patient appointments, and as such professionals were under time constraints. Several interviews had to be stopped and started again as the professionals needed to help patients or answer inquiries.

Before each interview began, interviewees were given information about the project and what participating would practically involve (the approximate length of the interview and the types of questions that the interview would cover). Interviewees were informed that the interviews would be tape-recorded, and that the interviews would be typed up and reported in an anonymous format. Respondents who completed face-to-face interviews were asked to give written consent to take part in the study. Respondents who completed telephone interviews were asked to give verbal consent.

The young adult interview schedule was divided into two sections. The first section asked young adults to talk about any diabetes-related issues that distressed them, and also day-to-day factors in their lives that they felt impacted their diabetes control. The second section of the young adult interview schedule asked young adults to talk about their perspectives on, and experiences of using, diabetes health services in Ireland; it is the data generated by these questions that is reported in this article. All young adult interviewees were asked the following open-ended questions:

•Can you tell me about your experiences of using diabetes health services in Ireland?

•What, if anything, are Irish diabetes services doing well for people your age?

•What, if anything, are diabetes services doing badly?

•Do you have any recommendations for improving Irish diabetes services?

The young adult interview schedule was piloted with two people with diabetes who were in their early thirties. The baseline interview questions did not change as the interviews progressed, though the interviewer probed issues that were raised in particular interviews, such as frustrations with the health system.

Healthcare professionals were asked questions similar to those asked of young adult interviewees, though with the wording of the questions were changed where appropriate (e.g. ‘what, if anything, are Irish diabetes services doing well for young adults with Type 1 diabetes in their twenties?’).

### Analysis

Interviews were thematically analyzed [21] in a word-processing package (MS Word). The first and the second authors double-coded the first four interviews and the other authors provided feedback on their analysis. The first author then analyzed the remainder of the interviews. Analysis for each interview began by ‘open coding’ the interview transcript, giving each section of the transcript that addressed a particular issue a descriptive tag or ‘code’. These codes were then compared and contrasted within and across interview transcripts in order to determine if some of them could be subsumed under high level concepts or ‘categories’ (e.g. all posts labelled with the descriptive tags ‘lack of continuity of care with professionals’ and ‘easy to access junior professionals/difficult to access specialists’ were placed under the higher level category ‘interactions with healthcare professionals’). The principle categories or themes that were identified from the interviews were: long waiting times; interactions with healthcare professionals; geographical inequity; cost-containment pressures; staffing issues; strategies used by young adults to obtain the best quality care; and satisfaction with services. Once these categories or themes were identified the authors searched the research literature for a conceptual framework that could best encapsulate them. Donabedian’s quality of care framework was determined to be suitable as most of the categories or themes that we identified could be fitted under Donabedian’s macro-concepts of ‘process’ (long waiting times, interactions with professionals) and ‘structure’ (geographical inequity, cost-containment pressures, staffing issues) [22-24]. One theme that we identified through coding, ‘obtaining good quality of care’, did not fit neatly into Donabedian’s framework but could be explained as patients’ responses to structural and process-related issues in the Irish diabetes health system. We took care to identify negative or contradictory cases, and highlight them here where relevant.

We checked the final draft of the article with the young adults who took part in the study. Young adults felt that the paper accurately described their experiences.

I’ve read over it is very interesting and very true. (Female, 26).

Had a quick read through and it seems excellent, I certainly wouldn’t have any discrepancies. (Female, 27).

### Ethical approval

The study received ethical approval from the Research Ethics Committee of the Royal College of Surgeons in Ireland.

## Results

Findings are presented in four sections. The first section discusses process-related issues in Irish diabetes services (waiting times, interactions with professionals, lack of communication from health services). The second section discusses the structural problems that professionals and young adults identified as applying to Irish diabetes services. The third section discusses the strategies that young adults used to obtain the best quality care in the Irish health system. The final section investigates young adults’ and professionals’ overall levels of satisfaction with Irish diabetes services.

### Process issues in the public system

#### Waiting times

Just under half of young adult interviewees (n=16) described long waiting times to see diabetes professionals. These young adults generally felt that they waited longer now to see healthcare professionals compared to when they were younger. Waiting times of between six months and a year – and very occasionally longer – were common. There appeared to be two reasons for this. One was that interviewees sometimes attended specialist young adult clinics when they were in their late teens and early twenties, which placed a strong emphasis on seeing young adults on a regular basis. Some of the interviewees in this study had transitioned out of these clinics and into general adult diabetes services where waiting times were much longer. However other young adults who had been treated in adult services for a number of years reported that waiting times in these services had lengthened since 2008, which they attributed to funding cuts in the Irish health service as a result of government austerity measures and insufficient numbers of specialist diabetes staff. Several professionals supported young adults’ assertions here.

At the start [when first diagnosed] the consultants were saying we want to see you back in three months, it might be a week or two weeks over. It wouldn’t be four or five months over the date. (Female, 28).

We’re down a diabetes nurse [because of the national recruitment embargo]. So already our waiting list has gone out a bit longer than we would have liked. (Diabetes nurse specialist, rural hospital).

Several young adults who reported long waiting times felt that they were ‘cursed for being good’, by which they meant that they felt that healthcare services were less concerned about them and therefore postponed their follow-up appointments as they demonstrated good control. These interviewees appeared to feel that Irish diabetes health services followed a triage philosophy, putting most of their efforts and limited resources into patients with poor control. In contrast, they felt that patients with good control tended to be forgotten, at least to an extent, by the system because their medical needs were not as pressing. However they felt that the criteria that healthcare professionals often appeared to use to determine time investment in patients (i.e. biological markers such as HbA1c), and the frequency with which they saw patients, were somewhat flawed as patients could have good control but still experience diabetes-related problems such as psychosocial distress.

I occasionally get the feeling that aren’t as worried about someone like me so they tend not to have me in as much. They even said to me at one point they want me in a year rather than six months time. (Male, 28).

You might go in and the doctors are trying to catch up with the backlog and if you’ve got no issues they’re just like, I’ll see you in three months time. That can be quite frustrating. (Female, 30).

Long waiting times were not limited to young diabetics seeking routine ‘check-ups’. A minority of interviewees (n=4) also described long-waiting times (up to a year) to be seen for urgent health problems such as blood vessel problems in their eyes.

I said I’m really ill, I said I need to see the consultant… she told me I was going to be waiting 18 months. (Female, 29).

Healthcare professionals agreed that young adults could experience long waiting times between appointments. However they also noted that some young adults contributed to this problem by not consistently turning up for their appointments and not calling clinics to reschedule. This meant that services were unable to reallocate these young adults’ time-slots to other patients and thereby reduce others’ waiting times. Professionals felt that the young adults of greatest concern – those with the worst HbA1scores and greatest psychosocial distress – were often the least likely to turn up for their appointments, though non-attendance was a significant problem for many young adults in their twenties. A number of young adults supported healthcare professionals here.

A factor that contributes to long waiting times is patients not coming to clinics. We usually have about 20-25% “no-shows”. (consultant).

There was a clinic last week with 30 people down to attend it, for age 18–30, only 12 showed. (Female 28).

The reasons why some young adults in this study did not turn up for their appointments varied but were often at least partly time-related. Young adults either felt that they did not have the time to go for their appointments (and particularly that they could not afford to take the time off work) or they felt that the appointments themselves were a ‘waste’ of time, for example because healthcare professionals would not have the time to discuss the issues that they wanted to discuss.

They say I can take x amount of hours out of my day whenever to come and sit in the hospital. Wrong. I can’t. (Female, 30).

Because of the lack of resources and specialist staff in the Irish public diabetes system (see next section), if a young adult missed an appointment it was often difficult to rebook them back into the system within an appropriate timeframe. As described by young adults and healthcare professionals, there was very little flexibility in many Irish diabetes services.

I know with the young person’s clinic if you miss one, the next one isn’t for another 6–12 months. Because the health service is so stretched it’s hard. (Female, 23).

Long waiting times negatively influenced interviewees’ diabetes management in three ways. They prevented interviewees from obtaining important information that they needed, such their HbA1c, to fine-tune their diabetes control. HbA1cC tests measure average blood sugar control over a three-month period. If the levels are tested yearly, then nine months of blood sugar information will be lost. Lengthy gaps between appointments also undermined interviewees’ motivation to optimally manage their diabetes. Many young adults noted that their enthusiasm for managing their diabetes often increased significantly in the weeks preceding and succeeding their appointments. The longer that interviewees went between appointments, however, the more their enthusiasm tended to ebb, and the more they began to skip blood sugar recording and neglect good eating practice. Thirdly, the longer young adults went between appointments the more that they needed to discuss with healthcare professionals. This meant that when they finally did get to see a healthcare professional there was often insufficient time to explore all of the items that needed to be talked about.

If you test HbA1c every six months means you’ve definitely lost something. You don’t know what was going on. They are not too concerned about that here (Male, 29).

For the first few weeks after clinic I’m all over it. But the longer you go between appointments the more things tend to drop. (Female, 28).

Finally, it is important to note while many young adults experienced problems accessing health professionals in a timely manner, the majority of young adult interviewees who took part in this study (n=19) indicated that they did not.

I don’t think I’ve had problems with waiting times. Not really, no. (Female, 27).

#### Lack of communication

Health service communication with young adults outside of clinical appointments was mixed. About half of young adult interviewees described good communication with health services, saying that health services were easily contactable and that services followed-up with young adults if they were experiencing difficulties with their diabetes. These services, mostly located in urban teaching hospitals, sometimes allowed young adults to upload their blood sugar results so that they could be provided with quick feedback.

I don’t think the care that I get could be better. If I ever have a problem or question I will email the nurse and get a reply from her the next day. ( Female, 27).

We interviewed healthcare professionals from these services. These professionals were satisfied with the service that they provided to young adults, but noted that providing good quality telesupport of this kind was a significant resource burden for them.

The picture for the rest of the young adults was more problematic. They described health services that had poor follow-up of patients and were difficult to contact. Interviewees who did attempt to contact these services looking for advice frequently received no response apart from the dial tone of an answering machine. They said that responses, when they did come, happened sometimes weeks later.

I’m sure any day now they’ll get in touch with me [sarcastic]. I’ve left messages but it being the Irish health service, I don’t expect anything. (Female, 30).

#### Interactions with healthcare professionals

About two thirds of young adult interviewees described sub-optimal interactions with diabetes healthcare professionals. Complaints about short consultation periods were commonplace, with interviewees commonly seeing healthcare professionals for no more than ten or fifteen minutes every six to twelve months. This was obviously not enough time to discuss young adults’ diabetes management concerns. Some professionals appeared to use the very limited time that they did have with young adults to go over biomedical or historical information that was of limited interest to young adults. Healthcare professionals themselves recognized that there was a significant problem here.

Instead of seeing Type 1 patients four times a year, we’re seeing them one time, very brief amounts of time and that’s inappropriate. (Consultant).

Maybe sometimes when you’re feeling a bit rushed and under pressure, you’re not giving the patients as much time as they would probably need to express themselves…[you’re just] going through all the tick boxes, what’s their HbA1c like and so on. (Nurse, rural hospital).

A significant proportion of these young adults’ care appeared to be provided by junior doctors with 1–3 years of experience and by registrars who were trainee specialists. These younger healthcare professionals were generally a source of frustration to young adult interviewees. Interviewees felt that junior doctors often did not know very much about diabetes, and that these doctors spent most of the consultation trying to learn from the patients themselves. When junior doctors provided advice, it was generally considered to be generic and basic when interviewees wanted detailed, personalised advice. Junior doctors rotated quickly through clinics so there were continuity of care issues here as well. Many interviewees did not see the same doctor from appointment to appointment. Discontinuous care was problematic not least because it disrupted professionals’ abilities to effectively provide advice to young adults. It was evident from the interviews that medical advice that was deemed by young adults to be threatening (most often advice which young adults felt was critical of their behavior) and which was provided by a stranger –irrespective of whether or not that stranger was a doctor or a nurse – was more likely to be rejected, or to make interviewees feel angry or distressed. In contrast, similar advice that was provided by a professional with whom the young adult had developed a positive relationship with was more likely to be considered and acted upon. Interviewees were unified in their desire to see consultants for their clinical appointments, but felt that it was often very difficult for them to access these senior professionals, particularly if nothing was ‘wrong’ with their diabetes control.

The last appointment really annoyed me because I’d never even seen this person before, she looked at my chart, asked me the same questions every single other person asks because they don’t know who you are. (Female, 23).

### Structure issues in the public system

#### Service inequity

Quality diabetes care was considered by many young adults and most healthcare professionals to be unevenly distributed throughout the country. If young adults had access to a specialist diabetes centre, for example in a large University teaching hospital, their probability of obtaining a good or excellent service was quite high. Professionals who worked in these services were considered to have strong interests in young adults with diabetes and were considered to be up-to-date in terms of their knowledge of diabetes medical technologies.

There’s no comparison between the care here in Dublin and what I would have experienced down the country. They’ve dietitians and doctors and nurses here and I’m seen regularly. (Female, 30).

Professionals felt that young adults who did not have access to specialist diabetes centres appeared to have a greater chance of receiving lesser quality of care.

I think if young adults can get into a specialty centre, I think it’s good. I think the service for anybody outside that small supply of good centres, I think it’s very poor. (Nurse, urban hospital).

Rural health services in particular often lacked a critical mass of specialist staff such as Endocrinologists specialising in diabetes. In some services nurses and more junior (non-consultant) doctors were mainly responsible for young adults’ diabetes care, and these professionals could be dealing with hundreds if not thousands of patients (Type 1 and Type 2).

We deal with 3000 people with diabetes and there’s one nurse. A lot of the rural hospitals like mine would be similar small units, minimal staff and a big patient population. (Nurse, rural hospital).

Rural services appeared to often be under-resourced meaning that they were unable to offer the diabetes education programmes that their urban counterparts could. Additionally diabetes technologies such as continuous subcutaneous insulin infusions(CSII,insulin pumps) were mostly concentrated in minority of clinics, meaning that young adults from outside these clinics’ catchment areas had fewer opportunities to obtain these devices. In order to obtain the best quality care a number of young adults from around Ireland were forced to attend diabetes clinics in Dublin or other large urban areas, which meant that they spent significant time and money travelling to their appointments.

One of the biggest stumbling blocks we came up against was finding a structured education programme for Type 1s. For a unit like ours that is minimally resourced we just don’t have that Type of funding. It’s not that we don’t want to do it, we’d love to do it. A lot of the rural hospitals would be similar. (Nurse, rural hospital).

We also only get 12 insulin pumps a year and it’s not necessarily the young adult that get them. We get 12 for the whole of the west of Ireland [primarily rural region]. (Nurse, urban hospital).

#### Cost-containment pressures in the Irish system

A number of young adults felt that the Irish healthcare system was increasingly concerned about providing them with the cheapest care rather than the best care. One young woman, for example, noted that auditors in the health system questioned her pharmacist to find out if she could be provided with fewer glucose test strips. A number of young adults also felt that pharmaceutical companies were beginning to fund functions that had previously been standard aspects of public sector care, such as glucometers and diabetes training programmes. About a quarter of young adult interviewees noted significant difficulties in obtaining diabetes technologies, particularly CSII and when they were offered these devices they were not given a choice about the model they could take. They felt that the health system restricted their ability to obtain the most advanced CSII devices that, although more expensive, would give them the best quality of life. One young woman who attended an urban teaching hospital in Dublin, however, noted that her consultant gave her a choice of three pumps from which to choose.

The chemist said do you need that many strips. I’m like, yeah. They’re like, there was a query about it. I’m hardly selling them on the street. (Female, 28).

You don’t get the best pump. You get the old one that costs less. (Female, 27).

I was on the list for the diabetes programme for 18 months. They just don’t have the resources. It’s a pharmaceutical company that are paying for it now. (Female, 30).

Healthcare professionals agreed that patients were often offered less expensive version of CSII but noted that many patients did well with such devices. One consultant noted that some of the more expensive CSII devices were intended for individuals who are unaware of low blood sugars rather than for all patients with diabetes. There was a discordance here between consultants and young adults, with some young adults believing that they were being denied CSII devices on cost grounds and consultants feeling that the more expensive pumps were unnecessary for some patients.

While acknowledging that cost-containment pressures in Irish diabetes health services were impacting on the care they could provide, consultant’s also noted positive aspects in that the health services still provided diabetic patients with free insulin, glucometers and diabetes-related medication. Several young adults agreed with this view.

I’d have to say I can’t complain at all. Everything is looked after. I’m not paying for anything. I get checks on my eyes, I can see a dietitian or podiatrist if I need to. We are very well looked after. (Male, 30).

#### Staffing issues

All interviewees felt that staffing problems – stemming from recent cuts in government funding to Irish health services –were an important cause of the process difficulties discussed previously.

It’s the constraints. They don’t have the money. I think it’s definitely money constraints. (Female, 26).

Many young adults described diabetes services using words such as ‘under-staffed’, ‘swamped’ and ‘struggling’.

One other thing I am aware of now in Ireland, unfortunately the hospital I’m going to for diabetes seems to be very understaffed. I get an appointment less than half the time that I got in the UK. (Male, 29).

I think due to budget cuts and stuff, staff started to get cut which was unfortunate but not their fault basically. (Female, 24).

Interviews with healthcare professionals indicated that diabetes healthcare services, in both urban and rural areas, often lacked sufficient numbers of consultant Endocrinologists and diabetes nurse specialists (this is one of the reasons why young adults were likely to be treated by junior doctors). The lack of Endocrinologists in the system was felt to be a historical legacy of the underfunding of diabetes services in Ireland. Consultants noted that the numbers of endocrinologists in Ireland at the time that the study was conducted (2012) was significantly below international standards. Both young adults and healthcare professionals felt that health services that lacked consultant Endocrinologists were at significant disadvantages.

The international and Irish recommendations is to have 5 to 6 consultant diabetologists per 250,000 population. There is no service in Ireland that has anywhere near that number. (Consultant).

We’re in an unfortunate position at the moment in that our endocrinologist retired a year ago. We don’t have anybody long-term to drive the service forward. (Nurse).

Some young adults and healthcare professionals felt, however, that the numbers of Endocrinologists in the system was beginning to slowly improve, albeit – as noted by the consultant in the previous paragraph – the overall numbers were still below international recommendations. However young adults who experienced consultant Endocrinologist-led care for the first time could experience dramatic improvements in the quality of care that they received.

We had no consultant endocrinologist until about 4–5 years ago. He came on board and he’s responsible for a lot of positive changes. (Female, 30).

The lack of diabetes nurse specialists in the system was also felt to stem from historical underfunding but was felt to be accentuated by recent cuts in funding as a result of the Irish government’s economic austerity programme.

The national embargo on public sector staff has affected the number of diabetes nurses. (Consultant).

A number of the nurses who we interviewed noted that diabetes nurses were not being replaced when they went on maternity leave, or were being replaced by non-specialist professionals, while at the same time services were dealing with increasing numbers of patients with Type 2 diabetes. Nurses felt that this problem – increased demand for diabetes services combined with a lack of capacity to meet that demand – has meant that services for people with Type 1 diabetes have begun to worsen in some parts of the country.

If there’s people going on maternity leave or being out of work for whatever reason they’re sometimes not replaced by people who are as competent. And that’s the recruitment moratorium. That’s definitely an issue. (Nurse).

B There shouldn’t be the restrictions that we have to deal with.

A Is that because of the funding constraints in the health service?

B It is absolutely. We don’t have the manpower or the equipment (Diabetes nurse specialist).

Services [for Type 1 patients] have kind of deteriorated in that we have so many Type 2s coming in and no extra staff. (Diabetes nurse specialist, urban University hospital).

There was also felt to be a significant lack of allied health professionals in the system.

I work as a dietician myself. Dietitian services are brutal [terrible]. (Female, 28).

Psychological services for young adults were considered to be especially lacking. This was despite the fact that all healthcare professionals considered psychological support to be an essential component of care for young adults with diabetes. Even the best service usually only had a psychologist on call for less than six hours a week- and that time was needed to cover all of the patients (Type 1 and Type 2) that the service treated. Healthcare professionals felt that the lack of psychologists in the system meant that many young adults with depression and eating disorders were going undetected, and when they were detected, were left untreated. Young adults who needed to see psychologists faced long-waiting times and the risk of being referred to different hospitals for treatment. Sometimes, if they were not at crisis point, they were forgotten. A number of nurses reported lobbying the Irish government for additional psychological resources, but noted that their entreaties fell on deaf ears.

A What’s your impression of psychological services?

B In Ireland?

A Yes.

B Oh awful. We have a psychologist six hours a week. It’s awful. (Nurse, urban hospital).

### Strategies for obtaining the best care in the Irish system

This section describes the strategies that young adults used to ensure that they obtained good quality care given the process and structure related issues outlined to this point.

A number of young adults were upset and confused about the quality of care that they were offered by the Irish system.

The system is crazy (Female, 28).

It makes me so angry. And nothing but cuts all the time. It kind of worries me that if I did end up in hospital with diabetes…no way. Sorry. That scares me. (Female, 30).

Sadly from what I have seen over the last few years, HSE (Irish health service) cuts mean ever fewer rather than more services for diabetes patients (Female, 27).

Many of these young adults were worried that the public health system did not care about them and would not look after them if they became seriously ill. Some felt that the only way to obtain good quality of care was to proactively obtain it. They used four main strategies to do this. One was to pay for private health care (either out of their own pocket or by obtaining private health insurance). For the interviewees who chose to do so (and there were quite a number of them), going private had a number of advantages: it enabled them to bypass waiting lists; it gave them access to health services that could often only be obtained privately, such as psychological counselling or chiropody services; it provided them direct access to consultants who they felt could provide them with better quality care and information than they would receive from junior doctors in the public system.

The consultant in x felt that I might have had a blood vessel problem and, again if I had gone publicly, the only appointment they could give me was 9 months. So we said straight away we’d go privately so we went to the x and saw Dr. x there. He did all the tests, everything, photographs, pupil pressure and everything was normal. (Female, 30).

Young adults noted a number of disadvantages with the private system, however. The main disadvantage was expense. A number of young adults also felt guilty about using the private system. They felt that doing so was morally problematic and unfair because other young adults did not have their resources. One interviewee who was considering purchasing private health insurance also described considerable struggles to find out what diabetes-related costs were covered by different insurance plans.

I even felt bad about going private because that’s not really the way you should do things. (Female, 28).

Ireland is full of a lot of shit and a lot of advertisement and at the end of it, what you get isn’t what they advertise. (Male, 29).

The second strategy was to demand good quality care from healthcare providers. Many of the young adults who we interviewed felt that unassertive patients would be neglected by the health system. Young adults stressed the importance of demanding to see consultants during appointments and of complaining to staff when they felt that they were receiving sub-standard care. In practice, however, interviewees often found it difficult to be this assertive. Some were worried about arguing with authority figures, others that if they complained they would be seen as ‘bad patients’ and healthcare professionals would punish them by providing them with lesser quality care. Embarrassment prevented a number of young adults from being assertive. Although young adults in this study were well in to their twenties, and some were around thirty, it was notable how frequently parents stepped in to advocate on behalf of their children.

Unless you actually say something they will forget about you. It’s crazy. Is it safe? No. It’s not. I started in the last year asking is the consultant here today, can I see him. I’m not leaving until I see him. Otherwise you could end up with a junior docto. (Female, 26).

My mum actually made an appointment privately with Dr. X. I probably wouldn’t have done it on my own. (Female, 28).

The third strategy was to transfer to another clinic/diabetes service, which usually proved to be difficult. There was often no objective information that young adults could use to determine if one clinic was better than another. As a result young adults who were considering transferring were often forced to rely on rumours or recommendations from friends or colleagues. Most searched for information on the Internet and found that clinics’ websites were quite basic. Understandably a number of them were reluctant to move clinics, possibly to a worse clinic, on the basis of a rumour.

A fourth strategy that young adults used was to develop diabetes services for themselves where these services were not forthcoming from the health system. For example, many young adults in this study wanted to receive emotional and social support to combat diabetes-related psychosocial distress. These young adults felt that the official diabetes system did not provide them with either the quality or the quantity of the support that they needed, and as a result of this they began to develop their own young adult peer support networks. We interviewed one young woman who established a peer support network. She felt that there was a great, and unmet, demand for peer support amongst young adults with Type 1 diabetes. Acknowledging this fact, she said that she went to her consultant to find out whether the health system could provide her with additional (and fairly minimal) resources to extend the peer support group that she developed. Her consultant was extremely enthusiastic about the idea, but said that the health system would not be able to fund it in any way.

I said to a consultant, ‘I know the Irish health system won’t want to know because it may cost money…’ He just laughed and was like, forget it. He said he can’t get what he needs and he is the hospital, not a support group. (Female, 30).

### Satisfaction with quality of care offered to young adults

For the reasons outlined in this article, most healthcare professionals considered quality of care for young adults with diabetes in Ireland to be unsatisfactory. Professionals felt that services were doing the best that they could be doing in a very challenging economic environment and that some specialist services were providing quality of care as good as anything that was offered internationally. However, they also felt that the Irish health service as a whole was not effectively meeting the needs of young adults with diabetes.

Services [for young adults with diabetes] are doing very poorly. (Nurse).

We’re talking about 20–30 year olds. I think the service we provide for Type 1 patients, no matter what age they are, is completely suboptimal. (Consultant).

In contrast, and despite the specific process and structural problems that they identified, most young adults indicated that they were satisfied with the quality of care that the Irish health system offered them. In fact, even a number of young adults who indicated that they were upset about the quality of Irish diabetes services also said that they were satisfied (at least to some extent) by those services.

At the moment I think the clinic is doing a very good job. Getting an appointment sometimes is a problem, but I think they’re pretty good to be honest. (Female, 28).

So why would a young adult be satisfied with services which he or she (and healthcare professionals) identified problems with? There were a number of reasons for this. The most common reason was that the young adult felt that they had established some kind of supportive personal relationship with a healthcare professional (most commonly a consultant endocrinologist or a diabetes nurse specialist) who worked in that service. Young adults often described this professional as going above and beyond what the young person thought was expected in terms of their duty of care, for example regularly telephoning the young adult to find out how their diabetes management was going, or providing them with intense, personalized instruction on the use of diabetes technologies, and so on. All young adults who indicated that they had established a supportive relationship with a professional expressed some degree of satisfaction with the diabetes health service in which that professional worked, even when they also identified significant problems with the service. Even young adults who rarely met their supportive professional often thought positively about the service; as long as a young adult believed that a supportive relationship existed, that relationship cast a positive halo effect around the service as a whole. This was the case even when the young adult described discontinuous interactions with other staff members in the setting, or other process related problems such as long waiting times.

X is my consultant there. It’s great that I regularly see him and I think the service is fantastic… the other doctors change and I know that’s to do with the medical system, the rotations but it’s really frustrating when you go in and you tell them the same thing every time. That really gets to me. (Female, 23).

There were also other reasons why young adults’ expressed satisfaction with services. A number of interviewees felt that even though they had problems, diabetes services were doing the best that they could given the difficult economic environment which they were operating within between 2008 and 2013. These interviewees noted the geographical differences in the quality of services, long-waiting times and staff shortages, but felt that responsibility for these problems lay with actors outside of diabetes services. As long as diabetes service appeared to be making an effort to meet their needs, these young adults felt satisfied.

I know they’re trying to do it as best they can. I think it’s good that even though things are quite bad [with the economy] at the minute, they manage to do the eye tests and they’ve brought in a podiatrist a couple of days a week. I think that’s good that they’re still taking a step in the right direction. (Female, 26).

Some interviewees, however, appeared to lack knowledge about what quality of care should be offered to them, for example being unaware of how often they should ideally see diabetes specialists or being unable to tell the difference between junior and senior doctors. This was problematic as it meant that young adults could accept suboptimal services without being aware that the care was suboptimal. Lack of awareness could also manifest itself in terms of misperceiving what services were available from young adults’ diabetes clinic. For example, one young woman noted that one of the things that she most readily valued about her clinic was that she could instantly access formal psychological support services if she should ever need them; whereas her consultant noted that the waiting list for psychological support in the service was a minimum of six months.

## Discussion

### Summary of findings

This is one of the few studies to investigate quality of care for young adults with Type 1 diabetes in their twenties. The study found that some Irish diabetes services appeared to be providing what young adults and healthcare professionals considered to be excellent quality of care. However many services seemed to experience a number of what Donabedian refers to as structural and process problems: long-waiting times, lack of communication between healthcare professionals and young adults; inadequate continuity of care; and over-reliance on junior doctors. High quality care also appeared to be unevenly distributed throughout Ireland, and rural services seemed to be at a marked disadvantage compared to their urban counterparts in terms of their ability to provide young adults with access to diabetes management technologies and structured education programmes. Young adults and healthcare professionals attributed many of these problems to staffing shortages in the diabetes health system, which in turn they often believed stemmed from funding cuts to Irish health services. A number of young adults responded the identified structural and process problems by proactively attempting to obtain high quality care, for example by obtaining private health insurance or by seeking to transfer to specialty diabetes clinics. Despite these problems, the majority of young adults expressed satisfaction with Irish diabetes health services. In contrast, healthcare professionals evaluated the Irish diabetes health system more negatively.

### Situating the results in relation to previous diabetes research

Several of the study’s findings have previously been reported (though the majority of this research has focused on young adults in the first phase of young adulthood). Previous studies have found that diabetes care is often problematic for young people with diabetes [5]. Their care can be discontinuous [25,26], which is of concern because discontinuous care can undermine the ability of healthcare professionals to meet young adults’ emotional, social and developmental needs [26]. Young adults prefer to meet Endocrinologists, diabetes nurse specialists and dieticians at their clinic appointments [27]. Young adults also place a great deal of value on professionals who take more ‘personal’ approaches and ‘get to know them’ [27]. This reflects wider understandings among patients- not just those with diabetes – that good quality care is based upon *caring*, and is patient focused and relationship-driven [28]. Our study suggests that many of these findings are as applicable to young adults in their late twenties as they are to young adults in their late teens. However whereas health services have put substantial efforts into improving quality of care for younger adults with diabetes (for example developing specialist young adult clinics that facilitate the development of continuity of care), the same efforts are often not put into the care of people with diabetes in their late twenties, who are often treated in undifferentiated adult services.

A new finding from this study is that some young adults feel that they are ‘cursed for being good’ – that health services are more likely to ignore them and/or delay their clinical appointments because they have good diabetes control. If this is indeed happening in some health services, this finding is very concerning. Health services cannot afford to relax their relationships with young people who have managed to achieve good control. Indeed, part of what helps young adults to achieve good control is having regular opportunities to talk to healthcare professionals. Not having regular appointments is likely to be a demotivating factor for at least some young adults and increase the likelihood that their control will become suboptimal.

### The Irish context

Many of the present findings stem from the particular context in which these young adults’ care was embedded. As noted by several healthcare professionals in this study, the healthcare system for young adults with Type 1 diabetes in Ireland has long been suboptimal, a state of affairs that stemmed at least in part from the historical underfunding of Irish diabetes services. This situation ameliorated somewhat during Ireland’s ‘Celtic Tiger’ years as more money was introduced into the Irish health system, including into diabetes services. The economic collapse that Ireland suffered as a result of its banking crisis, however, acted as an ‘external shock’ to the Irish health system, reducing funding again for structural aspects of diabetes care [29]. Cost containment became a key priority for the Irish health service, including diabetes services [29]. Government economic austerity measures led to embargos on the recruitment of specialist diabetes nursing staff and undermined some health services’ abilities to provide young adults with resources that they could use to improve their care, such as insulin pumps and diabetes education programmes. These structural issues (e.g. lack of specialist staff in the system) also contributed to the problematic process issues that some young adults reported experiencing, such as long waiting times to see healthcare professionals, suboptimal interactions with these professionals when young adults finally got to meet them and poor continuity of care.

However while Government austerity measures appear to be contributing to quality of care problems for young adults with diabetes, they are not the full story. The findings of this study suggest that austerity measures most pernicious effects seem to stem from their interactions with other care-undermining factors. One, as noted by some professionals, is the increasing numbers of patients with Type 2 diabetes who are coming into the Irish health system, which is reducing the time and resources that health services can put into the care of people with Type 1 diabetes of all ages, not just in their twenties. In effect, every Irish health service has a number of appointment slots that they can assign to patients. If services cannot increase the number of slots that they can offer to patients by hiring more staff, and they are dealing with enhanced demand for the slots that are available, services will be forced to offer individual patients fewer appointments. This is likely one of the main reasons why some young adults reported escalating time gaps between their appointments. Alternatively, some staff will feel that are forced to expend most of their time and efforts on the patients with the most serious distress and glycaemic problems, leading to an increased risk that young adults with better control and less emotional distress will receive less attention from health services, or more attention from less experienced staff. Young adults are themselves undermining their own quality of care, however, by not attending the appointments that are available to them. As noted by one consultant, a substantial minority of young adults in some clinics do not attend services. A number of young adults also reported that they regularly did not show up to diabetes clinics. By not attending services young adults are not only undermining their own care, they are undermining the care of other young adults who are struggling to access healthcare professionals’ limited time.

A number of young adults in this study responded to structural and process issues in the Irish health service by either attempting to develop their own services (i.e. support groups), by demanding care from professionals where they could do this or by seeking to escape into the private healthcare arena (and as noted, two-thirds of the young adults in this study had private health insurance). Young adults’ interest in private health insurance reflects a widespread belief amongst Irish people that individuals who pay to see medical professionals privately receive priority over public sector patients and can access health services more quickly (in what is often referred to as Ireland’s ‘two-tier system’) [30-32]. It also reflects a belief that private patients are more likely to be treated by consultants, whereas public patients are more likely to be treated by junior doctors [30]- which in fact seemed to be the case for some young adults in this study. Privatisation of care may be acting in some parts of Ireland as an escape valve for public sector difficulties, in that private services are fulfilling what should be essential elements of a good quality public system for young adults with diabetes. Some young adults feel that they have to pay to gain access to basic services (such as an opportunity to talk to a consultant, to get their feet examined etc.), and that libertarian, market-orientated principles [33] are a feature of Irish diabetes health services. This raises major issues around equity, where good quality healthcare can be seen as a commodity to be purchased by those who can afford it in an ‘age of austerity’, rather than as a right, as was enshrined in Ireland’s 2001 national health policy ‘Quality and Fairness’ which said that every patient in Ireland should experience fair access to a high standard of treatment.

### How good is quality of care for young adults with diabetes in Ireland?

A number of national and international recommendations outline the standards of care that should be offered to people with Type 1 diabetes, including young adults. The Irish Diabetes Expert Advisory Group [34] noted that all people with diabetes in Ireland should: receive care that is person-centred; be able to access care and education in a location that is convenient and acceptable to them; be able to access modern diabetes management technologies; be able to access psychological assessment and care. The American Diabetes Association [35] recommends that people with diabetes should have opportunities to participate in diabetes education programmes and should be screened for psychosocial distress. Specific guidelines for young adults with diabetes [1,4] have been developed that state that professionals need to develop individualised care plans for young adults, examine their psychosocial needs, assess their day-to-day barriers to diabetes management and establish continuity of care with them. These guidelines also recommend that healthcare professionals should ideally see young adults every three months [4]. Non-diabetes specific quality of care guidelines [36] indicate that good quality healthcare should increase the likelihood of desired health outcomes, be accessible, safe, patient-centred and timely and equitable (for example provide care that does not vary in quality because of geographic location, ability to pay etc.).

Assessed against these standards, quality of care for many, though by no means all, young adults with diabetes in Ireland appears to be problematic. This is perhaps not surprising; Evans et al. [37] note that in many parts of Ireland diabetes care seems to be provided in spite of the Irish health care system, rather than because of it. Many services, even specialist services, appear to be seeing young adults only once or twice a year. Care is often discontinuous from appointment to appointment. Many young adults are not offered easy access to diabetes education programmes or to diabetes management technologies, simply as a result of finding themselves attending the ‘wrong’ service in the ‘wrong’ part of the country. This rural–urban inequity (which may be a proxy for specialist centre-non-specialist centre inequity) is of concern, not least because geographic maldistribution of care is associated with poor outcomes in people with diabetes [38] and points to a phenomenon that has broader relevance internationally [39]. Bernard et al. [40] note that in an equitable system, resources should be equally accessible to all patients. In Ireland, resources for young adults with diabetes appear to be unequally distributed. Young adults outside of Dublin and other large urban regions such as Galway who do not have easy access to specialist diabetes centres appear to be at a significant disadvantage. This disadvantage is relative rather than absolute [41] (young adults outside of Dublin still have health services) but it is there. This is a failure to provide accessible care of the highest quality to all young adults. It is also a significant missed opportunity [9] given that good quality of care delivered at this time of life may have significant benefits for individuals with Type 1 diabetes.

### Potential solutions to quality of care problems

Addressing the identified quality of care issues could be done in several ways. One would be to introduce specialist young adult clinics to all diabetes health services, and enable young adults up to thirty years of age to attend these clinics. Another would be to transfer the care of uncomplicated patients with Type 2 diabetes to primary care, freeing up more specialized staff to work with patients with Type 1 diabetes. A third would be to ensure that senior Endocrinologists take a hands-on supervisory role in the care of all young adults with diabetes.

The truth is that most of the responses needed require additional resources, in a state where further significant cuts to the national health budget are being demanded by international lenders [42]. For example, there is a need for more Endocrinologist, diabetes nurse and allied health professional posts to be funded; and staff salaries, in Ireland as elsewhere, represent the greatest cost (circa 70%) of the national health budget. Caught, as specialist diabetes services in Ireland are, ‘between a rock and a hard place’, national policy makers will be faced by the need to manage difficult trade-offs in the different dimensions of quality of care. There is clearly a need to provide a comprehensive range of specialist services to young adults with diabetes, as they transition out of early adulthood, which will conflict in a resource-shrinking health system with the need to ensure accessibility for those living in rural areas.

### Satisfaction with services

One striking feature of young adults’ accounts was the high levels of satisfaction that they had with health services, even when they also identified problems with those services. Williams et al. [43] note that how patients arrive at an overall evaluation of health services can be complex, and that many expressions of satisfaction with health services can hide a variety of negative experiences. When users evaluate a service they are guided by beliefs about what the service should and should not do (what Williams et al. refer to as the perceived *duty* of the service) and whether or not the service is at fault if it is unable to do the things that it should do (what Williams et al. refer to as the *culpability* of the service). There may be only be a weak connection between an individuals’ experience of a service and how they evaluate it. In these terms, some young adults in this study felt that services were failing in their duty of care, for example by not providing easy access to consultants; though in other respects young adults felt that services (or individual members within those services) were exceeding their duty of care, for example by establishing supportive relationships with young adults. Even where services were believed to be failing in their duty, however, young adults mostly believed that these services were not culpable for these failings. Instead young adults often blamed the Irish government, or the more general international economic environment, for problems with Irish diabetes services.

### Strengths and limitations

The strengths of the study include a large qualitative sample and the inclusion of the perspectives of both patients and professionals. Limitations of the study include the fact that young adults took part in the study were clearly highly educated and economically advantaged (as demonstrated by their high levels of private health insurance); the majority of them also attended specialist settings in large urban areas and had reasonably good diabetes control. Despite their criticisms of Irish services, these young adults may have in fact been obtaining the best quality care that is available in Ireland. Saadine et al. [13] note that individuals with diabetes who do not have health insurance and have less education are more likely to experience worse quality of care [13]. It may well have been that if we had recruited more young adults from non-specialist settings (especially rural settings) who had less education and were more economically disadvantaged, the quality of care issues reported would have been worse. It must be noted as well that the majority of young adult interviewees were female. However gender did not seem to appear to have a significant impact on the Types of data that were generated by the project; most of the themes in this study (long waiting times, discontinuous care) would seem to applicable to all young adults, whether or not they are men or women.

### Additional articles from the same study

We have published three additional articles from this study. The first details our experiences of recruiting young adults through Facebook [44]. The second article [45] explores the experiences of young adults with Type 1 diabetes who have comorbid eating disorders, and the third [46] investigates causes of psychological distress in young adults with diabetes.

## Conclusion

This study investigated quality of care for young adults with diabetes in Ireland. It found that there were some excellent individual health services and diabetes-related policies in Ireland, such as the provision of free insulin and free diabetes medicine to young adult patients. However overall the findings are concerning and suggest that health system problems – accentuated by recent national austerity measures – are undermining quality of care for young adults with diabetes in Ireland. This will likely increase the risk of Irish young adults with diabetes experiencing negative long-term outcomes, with the attendant long-term cost-implications that will stem from such outcomes. Further quantitative research is needed to ascertain how representative the findings of this study are in the Irish context. More research that is non-specific to Ireland is needed on quality of care that is offered to young men with Type 1 diabetes in their late twenties, as well as to young people attending non-specialist settings those who are from disadvantaged backgrounds.

## Competing interests

The authors declared that they have no competing interests. This study was funded by a grant from Diabetes Ireland, the Medical Research Charities Group and the Health Research Board.

## Authors’ contributions

All authors were involved in the conceptualization and the design of the study. MB carried out the interviews. MB and FD analysed the interviews with SS, DS, RB and RC commenting on their analysis. MB and FD drafted the manuscript and SS, DS, RB and RC revised it. All authors read and approved the final manuscript.

## Pre-publication history

The pre-publication history for this paper can be accessed here:

http://www.biomedcentral.com/1472-6963/13/448/prepub
